# Beneficial immune modulatory effects of a specific nutritional combination in a murine model for cancer cachexia

**DOI:** 10.1038/sj.bjc.6604785

**Published:** 2008-11-18

**Authors:** J Faber, P Vos, D Kegler, K van Norren, J M Argilés, A Laviano, J Garssen, A van Helvoort

**Affiliations:** 1 Danone Research – Centre for Specialised Nutrition (formerly known as Numico Research), Wageningen, The Netherlands; 2 Cancer Research Group, Facultat de Biologia, Departament de Bioquímica i Biologia Molecular, Universitat de Barcelona, Spain; 3 Department of Clinical Medicine, University La Sapienza, Rome, Italy; 4 Department of Pharmacology and Pathophysiology, Utrecht Institute for Pharmaceutical Sciences (UIPS), Utrecht University, Utrecht, The Netherlands

**Keywords:** cachexia, immune function, inflammation, nutrition

## Abstract

The majority of patients with advanced cancer are recognised by impaired immune competence influenced by several factors, including the type and stage of the tumour and the presence of cachexia. Recently, a specific nutritional combination containing fish oil, specific oligosaccharide mixture, high protein content and leucine has been developed aimed to support the immune system of cancer patients in order to reduce the frequency and severity of (infectious) complications. In a recently modified animal model cachexia is induced by inoculation of C26 tumour cells in mice. In a pre-cachectic state, no effect was observed on contact hypersensitivity, a validated *in vivo* method to measure Th1-mediated immune function, after adding the individual nutritional ingredients to the diet of tumour-bearing mice. However, the complete mixture resulted in significantly improved Th1 immunity. Moreover, in a cachectic state, the complete mixture reduced plasma levels of pro-inflammatory cytokines and beneficially affected *ex vivo* immune function. Accordingly, the combination of the nutritional ingredients is required to obtain a synergistic effect, leading to a reduced inflammatory state and improved immune competence. From this, it can be concluded that the specific nutritional combination has potential as immune-supporting nutritional intervention to reduce the risk of (infectious) complications in cancer patients.

Cancer patients are recognised to be hampered by serious immune failures, especially patients with tumours of the head, neck, lung, oesophagus, cervix and breast (Pak *et al*, 1995; Mafune and Tanaka, 2000; Hadden, 2003). Several underlying mechanisms of immune dysfunction have been described to affect innate and adaptive immunity, leading to a poorer clinical outcome (Young, 1994; Young *et al*, 1996; Heimdal *et al*, 1999; Herber *et al*, 2007). The degree of immune dysfunction depends on the type and stage of the tumour, performance status, age, anti-tumour therapies, malnutrition and the presence of cachexia (Pak *et al*, 1995; Young *et al*, 2001; Argiles, 2005; Evans *et al*, 2006; Whiteside, 2006).

Cancer cachexia occurs in the majority of patients with advanced cancer. It is inversely correlated with the survival time of the patient and it always implies a poor prognosis (McNamara *et al*, 1992; Ross and Fearon, 2002; Zhou *et al*, 2003; Van Cutsem and Arends, 2005; Argiles *et al*, 2007). Cachexia is characterised by progressive, involuntary weight loss, wasting, anorexia, asthenia, fatigue and impaired immune function (Toomey *et al*, 1995; Puccio and Nathanson, 1997; Finley, 2000; Argiles, 2005; Van Cutsem and Arends, 2005). It has been estimated to account for 10–30% of cancer deaths, but might also contribute to deaths by other causes such as opportunistic infections (Warren, 1932; Inagaki *et al*, 1974; Barton, 2001).

Several mediators that are either tumour- or host-derived, such as pro-inflammatory cytokines, eicosanoids and hormones, have been implicated in the pathogenesis of cancer cachexia (Tisdale, 1997; Ross and Fearon, 2002; Argiles, 2005; Van Cutsem and Arends, 2005). The pro-inflammatory cytokines interleukin (IL)-1*β*, IL-6, tumour necrosis factor (TNF)-*α* and interferon (IFN)-*γ* (also called pro-cachectic cytokines) are thought to be responsible for the metabolic changes associated with cancer cachexia through different mechanisms (Toomey *et al*, 1995; Argiles *et al*, 2003; Van Cutsem and Arends, 2005; Morley *et al*, 2006). These cytokines each play a specific role, but it has become clear that overlapping biological activities and synergistic interactions between them lead to a progressive cachectic state (McNamara *et al*, 1992; Toomey *et al*, 1995; Argiles, 2005). An excessive amount of these cytokines, together with the major eicosanoid prostaglandin E_2_ (PGE_2_), lead to impaired immune responses that have been characterised *in vivo* by a progressive decrease in delayed-type hypersensitivity to recall antigens and to dinitrochlorobenzene (Stein *et al*, 1976; Haffejee and Angorn, 1979; Singh *et al*, 1979). Accordingly, a reduced *ex vivo* proliferation response of T lymphocytes to mitogens has been reported (Anderson *et al*, 1981; Wustrow and Issing, 1993; Hadden, 2003). Functionally, this leads to a higher susceptibility to infections in cachectic patients (Argiles, 2005; Ben-Baruch, 2006).

A good nutritional status is of major importance to maintain immune function in (pre-)cachectic cancer patients (Argiles, 2005). Nutritional interventions should therefore be recognised as an integral part of cancer therapy to improve clinical outcomes and quality of life (van Bokhorst-de van der Schueren, 2005). Early provision of nutritional support can stop or even reverse the decline in the nutritional status, and thus prevent the development of malnutrition and slowdown the progression of cachexia (Argiles, 2005; Correia *et al*, 2007). This is expected to lead to a better response to therapy and fewer treatment-related complications. Recently, a specific nutritional combination (SNC) has been developed to support the immune system of catabolic cancer patients before the onset of weight loss or already suffering from cachexia in order to reduce the frequency and severity of (infectious) complications. The SNC is based on four active nutritional ingredients, namely fish oil (FO), specific oligosaccharide mixture (SOM), high protein content and leucine (high protein/leucine), in which FO and SOM have been selected for their potential effects on the immune system.

Fish oil is a generally used ingredient in immune-modulating nutritional interventions, containing the conditionally essential long-chain n-3 polyunsaturated fatty acids (PUFAs) eicosapentaenoic acid (EPA) and docosahexaenoic acid (DHA). These long-chain n-3 PUFAs possess a wide range of anti-inflammatory activities, including a decreased production of the inflammatory mediator PGE_2_ and the pro-inflammatory cytokines TNF-*α*, IL-1*β*, IL-6 and IL-8 (Calder, 2006). In tumour-bearing rats, n-3 PUFAs prevented the decrease in body weight (BW) because of cachexia, and as a consequence, survival was increased significantly (Pizato *et al*, 2006). Clinical evidence of immune-modulating activities of long-chain n-3 PUFAs exists (van Bokhorst-de van der Schueren, 2005), however, generally without affecting systemic immune biomarkers (Sijben and Calder, 2007). The optimal dose, formulation, relative contributions of EPA and DHA in fish oil and the target population remain yet to be defined (Fearon *et al*, 2006; Sijben and Calder, 2007). The use of non-digestible carbohydrates, especially of prebiotic oligosaccharides, is based on the immune-modulating activities observed in several animal experiments and clinical trials (Vos *et al*, 2007). In an influenza vaccination model in healthy C57BL/6 mice, a specific mixture of oligosaccharides stimulated the vaccine-specific delayed-type hypersensitivity response as a marker for T-helper 1 (Th1) immunity (Vos *et al*, 2006). Enhancing systemic Th1-dependent adaptive immune responses would lead in theory to better immune responses against infections that have been confirmed by applications in clinical trials (Bruzzese *et al*, 2006; Arslanoglu *et al*, 2007).

The addition of high protein and the branched chain amino acid leucine to the product focuses on alterations in protein metabolism in cancer patients and might in combination with fish oil have a positive effect on skeletal muscle function, lean body mass and daily activity (unpublished data).

In this study, the effects of the individual nutritional ingredients, FO, SOM and high protein/leucine, were investigated on inflammatory status, immune function and on different parameters for cachexia. In addition, the effect of the complete mixture of these nutritional ingredients was tested and compared with the individual ingredients. The experiments were performed using a recently modified and validated animal model for cancer cachexia, based on the colon-26 tumour model, in which parameters of immune function can be quantified. In this model, cachexia is induced by the inoculation of murine colon adenocarcinoma (C26) cells in syngenic CD2F1 mice (Tanaka *et al*, 1990), leading to several cachectic features (Strassmann *et al*, 1992a; Tanaka *et al*, 1993; Soda *et al*, 1994). The model was extended by including several immune parameters: contact hypersensitivity (CHS) against oxazolone was measured in a pre-cachectic state as an *in vivo* parameter for Th1-mediated immune function. In addition, several *ex vivo* parameters were measured at a later time point, when the animals were in a cachectic state. These pre-clinical experiments were performed to evaluate the potential benefits of specific nutritional interventions for immune function, which might lead to new applications for cancer patients.

## Materials and methods

### Animals and diets

Six to seven-week-old syngenic male CD2F1 mice (BALB/c × DBA/2) were obtained from Harlan Nederland (Horst, The Netherlands). All experimental procedures were approved by the Animal Experimental Committee and complied with the principles of laboratory animal care. Animals were housed individually in a climate-controlled animal care facility with a constant room temperature and humidity. All animals had free access to food and drinking water. Upon arrival, animals were acclimatised for 1 week and subsequently randomised on the basis of BW. The experiments were divided into: experiments A, designed to test the effect of the individual ingredients, and experiments B, designed to test the effect of the complete mixture of ingredients that resembles the composition of the new generation FortiCare (Nutricia Advanced Medical Nutrition, Zoetermeer, The Netherlands). In both experiments A and B, mice were divided into a control group (C) receiving control diet, a tumour-bearing control group (TB) receiving control diet and tumour-bearing experimental groups (TB-nutritional ingredient). Data shown are derived from the combination of several experimental runs with identical animal characteristics and experimental procedures (unless stated otherwise).

The tumour-bearing experimental group in the experiments A received a diet based on AIN93-M (Research Diet Services, Wijk bij Duurstede, The Netherlands) with fish oil (TB-FO), SOM (TB-SOM) or high protein enriched with leucine (TB-HPrleu) supplied as pellets, and in the experiments B, a diet with the combination of fish oil, SOM and high protein/leucine (TB-SNC). The latter diet differed in the macronutrient composition from AIN93-M to achieve a more humanised diet supplied as dough for product technical reasons.

The control diet in the experiments A contained per kg food: 126 g protein (100% casein), 727 g carbohydrates and 40 g fat (100% soy oil). The experimental diets in the experiments A were adapted by adding 22.1 g fish oil (providing 6.9 g EPA and 3.1 g DHA) per kg food (TB-FO), 18 g short-chain galacto-oligosaccharides (Vivinal GOS, Friesland Domo Foods, Zwolle, The Netherlands) and 2 g short-chain fructo-oligosaccharides (Beneo p95, Orafti, Wijchen, The Netherlands) per kg food (TB-SOM) or 151 g casein per kg and 16 g leucine per kg food (TB-HPrleu).

The control diet in the experiments B contained more fat, although the diet is isocaloric and isonitrogenous compared with the control diet in experiments A, per kg food: 126 g protein (100% casein), 699 g carbohydrates and 52.6 g fat (100% corn oil). This control diet did not show any effect on physiological cachexia parameters and immune parameters in the used animal model (data not shown). The experimental diet in experiments B contained per kg food: 210 g protein (189 g intact protein of which 68% casein and 32% whey and 21 g free leucine), 561 g carbohydrates, 52.5 g fat (20.2 g corn oil, 10.2 g canola oil and 22.1 g fish oil (providing 6.9 g EPA and 3.1 g DHA)), 18 g short-chain galacto-oligosaccharides and 2 g short-chain fructo-oligosaccharides.

### Experimental design

Murine colon-26 adenocarcinoma cells were used to induce cachexia in mice (Tanaka *et al*, 1990; Strassmann *et al*, 1992a, b). In short, on day 0, tumour cells (5 × 10^5^ cells in 0.2 ml) were inoculated, under general anaesthesia (isoflurane/N_2_O/O_2_), subcutaneously into the right inguinal flank of CD2F1 mice in the tumour-bearing groups. Animals in the control group received a sham injection with 0.2 ml HBSS. Body weight, food intake and tumour size (length and width) were measured three times a week. To investigate effects on the immune system, CHS against oxazolone was determined, as an *in vivo* model for cellular (Th1-dependent) immunity. Briefly, on day 8, all animals were sensitised with 150 *μ*l 3% oxazolone solution (4-ethoxymethylene-2-phenyl-2-oxazolin-5-one; Sigma-Aldrich Chemie, Zwijndrecht, The Netherlands; 300 mg in 7.5 ml 96% ethanol and 2.5 ml acetone) applied on their shaved breast and abdomen. Subsequently, at day 13, ear thickness was measured under general anaesthesia and all animals were hapten challenged with 25 *μ*l 0.8% oxazolone solution (32 mg in 3 ml 96% ethanol and 1 ml acetone) topical to the ear pinnae. At day 14 after tumour inoculation (24 h after the challenge), ear swelling was measured under general anaesthesia to determine the Th1 immune response.

At day 20, blood was collected by cardiac puncture and sampled in heparin tubes. After killing, spleens were dissected, weighed and stored in a cold culture medium (RPMI-1640 containing 25 mM HEPES and 2 mM L-glutamine; Life Technologies, Merelbeke, Belgium; enriched with 100 U ml^−1^ penicillin/streptomycin) with 10% heat-inactivated foetal calf serum (FCS^hi^) for immunological analysis. Skeletal muscles (m. tibialis anterior (mTA), m. extensor digitorum longus (mEDL), m. soleus (mS) and m. gastrocnemius (mG)), tumour, epididymus fat and thymus were dissected, weighted and frozen at −80 °C (skeletal muscles).

### Immunological analysis

All *ex vivo* incubations of cells were performed at 37 °C in a humidified environment containing 5% CO_2_.

#### Whole blood assay

Blood was added in 50 *μ*l/well to 100 *μ*l/well culture medium in a 96-well plate and was subsequently incubated with 50 *μ*l/well LPS (Fc 1 *μ*g ml^−1^, *Escherichia coli*, B55:O55; Sigma-Aldrich Chemie) or culture medium (control) for 20 h or with ConA (Fc 40 *μ*g ml^−1^, concanavalin A from *Canavalia ensiformis* type IV; Sigma-Aldrich Chemie) or culture medium (control) for 44 h. Afterwards, supernatants were harvested and stored at −80 °C until analysis. Plasma was obtained from the residual blood and stored at −80 °C until analysis.

#### Splenocyte assay

Splenocytes were isolated by pressing spleens through cell strainers (40 *μ*m) into a 50 ml tube. After erythrocyte lysis, splenocytes were plated in 20 *μ*l per well (Fc 2 × 10^5^ cells per well) in a 96-well plate and 80 *μ*l per well culture medium with 10% FCS^hi^ was added. Thereafter, cells were incubated with 100 *μ*l/well LPS (Fc 1 *μ*g ml^−1^) or culture medium (control) for 20 h for PGE_2_ and cytokine production or with 100 *μ*l/well ConA (Fc 3 *μ*g ml^−1^) or culture medium (control) for 44 h both for proliferation and cytokine production. After 28 h incubation, cells for proliferation were labelled with 0.4 *μ*Ci/well tritiated thymidine (^3^H-thymidine; Perkin Elmer, Zaventem, Belgium) and incubated for another 16 h. Subsequently, cells were harvested on Packard GF/C filter plates (Perkin Elmer) and dried on air. Afterwards, 25 *μ*l scintillation fluid (Packard Ultima Gold; Perkin Elmer) was added to the wells and plates were counted in a scintillation counter (Wallac MicroBeta radioactivity plate counter, Perkin Elmer). For PGE_2_ and cytokine production, plates were centrifuged and supernatants were harvested and stored at −80 °C until analysis.

#### PGE_2_ and cytokine measurement

Prostaglandin E_2_ was measured using a commercial anti-PGE_2_ rabbit polyclonal antibody-based direct enzyme immunoassay (Oxford Biomedical Research, Oxford, MI, USA) according to the manufacturer's protocol. Cytokines in plasma were measured using a commercial mouse cytokine 10-plex bead immunoassay (Biosource, Etten-Leur, The Netherlands) according to the manufacturer's protocol, and cytokines in supernatants were measured using a commercial mouse cytokine Th1/Th2 bead immunoassay or a commercial mouse cytokine inflammatory bead immunoassay (both from Biosource) according to the manufacturer's protocol.

#### Flowcytometric analysis

Splenocytes were added in 100 *μ*l to a 96-well plate (Fc 1 × 10^6^ cells/well), centrifuged and resuspended in cold PBS with 1% FCS^hi^ and 0.1% sodium azide. Cells were incubated with fluorescent-labelled antibodies for 30 min on ice in a total volume of 50 *μ*l. After washing, cells were analysed on an Epics XL flowcytometer (Beckman Coulter, Mijdrecht, The Netherlands). The following monoclonal antibody combinations were used: (1) GR-1-FITC (BD Pharmingen, Alphen aan den Rijn, The Netherlands), F4/80-PE (Serotec Ltd., Oxford, UK) and 7-AAD (Coulter Immunotech, Beckman Coulter); (2) CD3-FITC, CD4-PE-CY5 (both from BD Pharmingen) and CD8-PE (Beckman Coulter); (3) TCR-alpha-FITC, DX5-PE and CD19-PE-CY5 (all from Beckman Coulter) and (4) unlabelled cells. Results were analysed using Expo32 software (Beckman Coulter). The method to determine positive cells using these antibodies was validated using isotype control antibodies in earlier experiments. Dead 7-AAD^+^ cells were excluded in antibody combination (1). The forward- and side-scatter profile from 7-AAD^−^ cells in combination (1) was used to identify living cells and exclude dead cells in combinations (2), (3) and (4).

#### Determination of phospholipid fatty acids in blood cells and splenocytes

Residual heparin blood and 1 × 10^7^ splenocytes were centrifuged and cell pellets were stored at −80 °C until analysis. Phospholipids were separated from total cellular lipids using Bond-Elut® solid-phase extraction columns and the Vac-Elut SPS 24™ system. Phospholipid extracts were converted into methyl esters by using 10% BF_3_ in methanol at 100 °C for 60 min. After hexane extraction, derivatised phospholipids were dissolved in iso-octane, and the fatty acid composition was analysed by gas chromatography using a capillary column (50 m × 0.25 mm, CP-SIL88-fame). Peaks were identified by commercial reference standards.

#### Statistics

**Figure 1:**
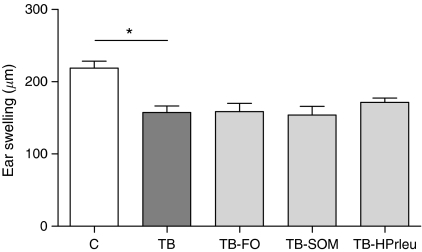
Effects of oral administration of fish oil, specific oligosaccharide mixture or high protein/leucine on contact hypersensitivity. Data represent means (*μ*m)±s.e.m. of the control (C) group (*n*=20), tumour-bearing control (TB) group (*n*=20) and tumour-bearing groups after supplementation with fish oil (TB-FO, *n*=10), specific oligosaccharide mixture (TB-SOM, *n*=10) or high protein/leucine (TB-HPrleu, *n*=10). ^*^Significantly different (*P*<0.0125) from the TB group.

**Figure 2:**
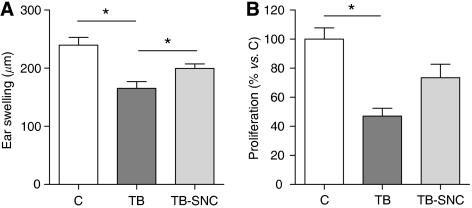
Effects of oral administration of the complete mixture of fish oil, specific oligosaccharide mixture and high protein/leucine on contact hypersensitivity (**A**) and ConA-stimulated T-lymphocyte proliferation by splenocytes (**B**). Data represent means±s.e.m. of the control (C) group (*n*=10), tumour-bearing control (TB) group (*n*=19) and tumour-bearing group after oral administration of the specific nutritional combination (TB-SNC) (*n*=20). For ConA-stimulated T-lymphocyte proliferation, all values were calculated as the percentage of the control group, which is set at 100%. ^*^Significantly different (*P*<0.025) from the TB group.

**Figure 3:**
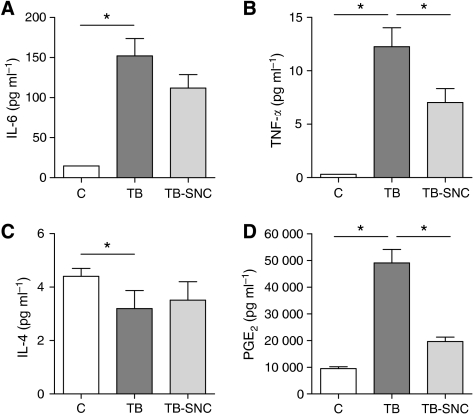
Effects of oral administration of the complete mixture of fish oil, specific oligosaccharide mixture and high protein/leucine on plasma cytokine IL-6 (**A**), TNF-*α* (**B**), IL-4 (**C**) and PGE_2_ (**D**) concentrations. Data represent means (pg ml^−1^)±s.e.m. of the control (C) group (*n*=10), tumour-bearing control (TB) group (*n*=19) and tumour-bearing group after oral administration of the specific nutritional combination (TB-SNC) (*n*=20). ^*^Significantly different (*P*<0.025) from the TB group.

All data were expressed as means±s.e.m. Statistical analysis was performed using SPSS 12.0.1 (SPSS Benelux, Gorinchem, The Netherlands). In the experiments A, different batches of animals were used. For that reason, it was examined for all parameters, by using ANOVA, if combination of the data was allowed and if no interaction between the groups and experiments existed. The effect of treatment was tested using a one-way ANOVA, followed by LSD *post hoc* analysis when data were normally distributed and showed equal variances. When equal variances were not assumed, *post hoc* a Dunnett's T3 test was performed. A non-parametric Mann–Whitney *U*-test was performed when data were not normally distributed. Differences were considered significant at *P*<0.0125 (Table 1 and Figure 1) and *P*<0.025 (Tables 2 and 3 and Figures 2 and 3) based on *α*/*k*, in which *α*=0.05 and *k* is the number of comparisons.

## Results

### Functional parameters measured in both experiments A and B

#### Physiological cachexia and immune parameters

At day 20 after tumour inoculation, mice were killed and both cachexia and immune parameters were measured. Data from the different experiments with the individual nutritional ingredients (experiments A) were combined and displayed in Table 1, and data from the experiment to test the efficacy of the complete mixture of FO, SOM and high protein/leucine (experiment B) were presented in Table 2. In experiments A, BW and carcass weight (CW=BW−tumour weight (TW)) were significantly decreased from 24.4 g (both) in the control (C) group to 22.8 and 20.7 g, respectively, in the TB group, whereas in experiment B, a decrease was observed from 25.7 g (both) in the C group to 20.1 and 18.0 g, respectively, in the TB group. This reduction could be caused by the significant weight loss of fat and skeletal muscles in the TB group. Food intake has been controlled and was not affected in both experiments A and B (data not shown).

The addition of one of the individual nutritional ingredients to the diet did not result in any significant effect on BW or CW compared with animals in the TB group. However, a diet containing the complete mixture of FO, SOM and high protein/leucine improved both BW and CW significantly from 20.1 and 18.0 g, respectively, in the TB group to 21.9 and 20.3 g, respectively, in the TB-SNC group (Table 2), indicating a less cachectic state of the mice. This was emphasised by a positive effect on other cachectic features, such as a significant inhibition of weight loss of epididymus fat and skeletal muscles, which was absent after feeding a diet with the individual nutritional ingredients.

In both experiments A and B, thymus weight was significantly decreased after tumour inoculation with 47.9 and 61.7%, respectively, whereas spleen weight was more than twice as high in the TB group as compared with the C group. After the addition of FO or the complete mixture of FO, SOM and high protein/leucine to the diet, a significant inhibition of thymus weight loss was observed, whereas none of the individual nutritional ingredients affected spleen weight (Table 2).

#### CHS

A CHS test was performed at day 13/14 to determine *in vivo* immune function prior to weight loss. Contact hypersensitivity responses were significantly reduced in the TB group as compared with the C group in experiments A (28.1%, Figure 1) and B (31.0%, Figure 2a), indicating an impaired Th1 immune response in tumour-bearing mice. After adding one of the individual nutritional ingredients to the diet of tumour-bearing mice, no effect was observed on this immune biomarker (Figure 1). However, after administration of the complete mixture of FO, SOM and high protein/leucine to the tumour-bearing mice (TB-SNC), immune responsiveness was increased significantly by 20.7% compared with the TB mice, indicating a better Th1-mediated immune response (Figure 2a).

### Additional immune parameters measured in experiment B

To obtain more information on immunocompetence and inflammatory status of tumour-bearing mice in a cachectic state and to determine the effect of adding the complete mixture of FO, SOM and high protein/leucine to the diet of these mice, several additional immune parameters were measured after killing the mice at day 20.

#### Flowcytometric analysis of splenocytes and determination of phospholipid fatty acids

Spleen weight was increased significantly in tumour-bearing control animals because of an increase in the number of spleen cells caused by an enormous expansion of granulocytes from 50.8 × 10^7^ cells/spleen in the C group to 598.9 × 10^7^ cells/spleen in the TB group (Table 2). In addition, monocytes showed a significant increase in the number of cells per spleen, whereas the total number of CD3+CD4+ and CD3+CD8+ T cells was not affected. Relatively, the percentages of CD3+CD4+ and CD3+CD8+ T cells were almost two times lower in the TB group compared with the C group, probably due to the high increase of granulocytes. After adding the complete mixture of nutritional ingredients to the diet of tumour-bearing mice, no effect was observed on the total number of splenocytes, neither on the absolute nor on relative number of granulocytes, monocytes and CD3+CD4+ and CD3+CD8+ T cells.

In cell membranes of the isolated splenocytes, percentages of n-6 and n-3 fatty acids of total phospholipid fatty acids were measured (data not shown). No effect was observed on the total n-6 fatty acids content after tumour inoculation, whereas the percentage of total n-3 was increased significantly in the TB group, probably due to the significant increase in DHA. After feeding a diet with the total combination of the nutritional ingredients, total n-6 content in cell membranes of splenocytes was decreased significantly compared with the TB group because of a strong reduction in arachidonic (AA) acid from 19.6% in the TB group to 8.9% in the TB-SNC group. The percentages of the n-3 fatty acids EPA and DHA were increased significantly from 0.4 and 2.5%, respectively, in the TB group to 3.5 and 4.4%, respectively, in the TB-SNC group, leading to a significantly higher n-3 content.

#### Plasma cytokine and PGE_2_ concentrations

Pro-inflammatory cytokines and PGE_2_ were measured in plasma obtained from blood at day 20. IL-6, TNF-*α* as well as PGE_2_ were increased significantly from 14.8, 0.3 and 9488 pg ml^−1^, respectively, in the C group to 152.1, 12.3 and 49 096 pg ml^−1^, respectively in TB group (Figures 3a–c), whereas IL-1*β* and IFN-*γ* levels were below the detection limit of the assay. A strong decline in the production of the pro-inflammatory cytokine levels, IL-6, TNF-*α* and PGE_2_, was observed at 111.8 (*P*=0.038), 7.0 (*P*=0.017) and 19 601 pg ml^−1^ (*P*<0.001) (all one-tailed tested), respectively, after the addition of the complete mixture of FO, SOM and high protein/leucine to the diet of tumour-bearing mice, leading to a lower inflammatory state.

#### *Ex vivo* ConA-stimulated T-lymphocyte proliferation in splenocytes

In addition to CHS, which was measured prior to weight loss, *ex vivo* ConA-stimulated T-lymphocyte proliferation by splenocytes was measured in cachectic tumour-bearing mice. A significant decrease was observed from 100% in the C group (all values were calculated as percentage of the control group, which is set at 100%) to 47.0% in the TB group (Figure 2b). After adding the complete mixture of nutritional ingredients to the tumour-bearing mice, a strong trend to an improved immune response was observed, although this effect was not significant (*P*=0.031).

#### *Ex vivo* ConA and LPS-stimulated cytokine production in whole blood and splenocytes

In ConA-stimulated whole blood and splenocytes, both Th1 and Th2 cytokines were measured, whereas in LPS-stimulated whole blood and splenocytes, pro-inflammatory cytokines and PGE_2_ were measured. All values were calculated as the percentage of the control group, which is set at 100%. In ConA-stimulated whole blood, IL-4 (Th2 cytokine) production was reduced significantly from 100% in the C group to 11.3% in the TB group (Table 3). Moreover, in ConA-stimulated splenocytes, IL-4 (Th2) as well as IL-2 and IFN-*γ* (Th1) were decreased significantly in the TB group. After administration of the complete mixture of FO, SOM and high protein/leucine to the tumour-bearing mice, IL-4 production in both ConA-stimulated whole blood and splenocytes showed a significant increase (*P*<0.025).

In LPS-stimulated whole blood, both IL-1*β* and TNF-*α* were decreased significantly from 100% in the C group to 17.5 and 18.5%, respectively, in the TB group (Table 3). In addition, a trend to decreased IL-6 levels was observed, whereas PGE_2_ showed a trend to enhanced levels in the TB group. In LPS-stimulated splenocytes, IL-6 was reduced significantly in the TB group compared with the C group. By contrast, PGE_2_ production showed a significant increase from 100% in the C group to 178% in the TB group. After feeding a diet with the complete mixture of nutritional ingredients, PGE_2_ production in LPS-stimulated whole blood was decreased significantly from 137.9% in the TB group to 43.1% in the TB-SNC group, whereas PGE_2_ production in LPS-stimulated splenocytes was not affected.

## Discussion

This study showed a significant improved Th1 immune response after feeding a diet containing the complete mixture of nutritional ingredients FO, SOM and high protein/leucine in tumour-bearing animals in a pre-cachectic state. In addition, in mice already suffering from cachexia, the complete mixture of ingredients affected several physiological and immune parameters, representing a lower inflammatory state, better immune responses and less wasting of protein and lipid stores, leading to less severe cachexia.

The cachectic features of the C26 mouse model have been described earlier by other authors, who attributed a pivotal role for the pro-inflammatory cytokine IL-6 in the induction of cachexia (Tanaka *et al*, 1990; Strassmann *et al*, 1992a; Tanaka *et al*, 1993; Soda *et al*, 1994). In the current study, parameters of immune competence were measured in parallel to cachexia- and inflammation-related parameters, to study cachexia-related immune dysfunction in this model as well. To this end, *in vivo* Th1-related immune function was quantified using oxazolone-induced CHS responses. In addition, several *ex vivo* parameters were measured in whole blood and isolated splenocytes cultures.

In both experiments A and B, BW and CW were reduced significantly in the TB group, which is in accordance with a significant reduction in the weight of epididymus fat and skeletal muscles and argues for a cachectic status of the mice. In experiment B, the pro-inflammatory cytokines IL-6 and TNF-*α* were measured. These cytokines have been described as pro-cachectic cytokines involved in different metabolic changes associated with wasting during cancer cachexia (Toomey *et al*, 1995; Argiles *et al*, 2003; Argiles, 2005). In literature, TNF-*α* levels measured in the C26 model were below the detection limit (Strassmann *et al*, 1992a; Soda *et al*, 1994). In this study, however, TNF-*α* levels can be measured, in spite of the low levels, and differences between groups were determined. Both IL-6 and TNF-*α* levels were increased significantly in the TB group, which confirmed the role of TNF-*α* besides IL-6, in the pathogenesis of cancer cachexia in the C26 model. By contrast, levels of IL-4, in literature stated as an anti-cachectic cytokine (Argiles *et al*, 2003), showed a significant decrease in the TB group, which is in agreement with the cachectic state of the animals.

Contact hypersensitivity responses were measured in a pre-cachectic state and were reduced significantly in the TB group, indicating an impaired Th1 immune response in tumour-bearing mice before the onset of weight loss. In earlier experiments, CHS was also determined in mice already suffering from cachexia, showing an even stronger reduction (unpublished data). It should be realised that this reduction was severe because of the dramatic health status of the mice at this time point, therefore the effect of the nutritional ingredients was only determined in a pre-cachectic state. Nevertheless, in cachectic mice, *ex vivo* parameters were measured to assess immune competence.

To evaluate the potential benefits of specific nutritional interventions on cachexia features and immune function, FO, SOM and high protein/leucine were added to the diet of tumour-bearing mice. No effect of the individual ingredients was shown on BW, CW, epididymus fat weight and weight of skeletal muscles, indicating no advances in the poor cachectic state of the mice. This was confirmed by the absence of potential effects on immune function, measured by CHS in a pre-cachectic state. When FO in combination with high protein/leucine was added to the diet, BW, CW and weights of epididymus fat and the skeletal muscle mTA were improved significantly (unpublished data). Moreover, CHS responses appear to show a small increase, but this was far from significant (*P*=0.716) compared with animals in the TB group (data not shown). However, after the addition of the complete mixture of FO, SOM and high protein/leucine to the diet of tumour-bearing animals, a significant increase in ear swelling was observed compared with those in the TB group, indicating an improved Th1 immune response leading to a better resistance against infections. In addition, in mice already suffering from cachexia, physiological parameters as BW, CW and weights of epididymus fat and skeletal muscles showed a significant increase after adding the complete mixture of nutritional ingredients to the diet compared with the reduction observed in TB mice (Table 2). Consequently, the cachectic state of the mice was reduced significantly, and muscle function and daily activity, important contributors to the quality of life, were improved significantly (unpublished data). Taken together, the results indicate that the combination of the nutritional ingredients FO, SOM and high protein/leucine is able to induce a synergistic effect, leading to less severe cachexia and improved immune responses.

Thymus weight was decreased after tumour inoculation and showed a significant recovery after adding the complete mixture of FO, SOM and high protein/leucine to the diet. The thymus is one of the central primary lymphoid organs and plays an important role in cellular immunity by generating circulating T lymphocytes (Delves and Roitt, 2000). Therefore, the recovery of thymus weight is of major importance for an efficient working and balanced immune system. In contrast to thymus weight, spleen weight was elevated in the TB group. This is mainly caused by a strong increase in both percentage and total number of granulocytes among the total splenocytes population. In literature this is known as a leukemoid reaction, which is earlier described in tumour-bearing mice and in different types of human cancers (Nimieri *et al*, 2003; DuPre and Hunter, 2007). When the complete mixture of FO, SOM and high protein/leucine was added to the diet of tumour-bearing animals, the leukemoid reaction was not reduced. Therefore, the beneficial effect of nutritional intervention on immune function is argued to be due to other effects on the immune system.

In addition to the measurement of CHS, several *ex vivo* assays were performed to determine immune function in cachectic mice. In ConA-stimulated splenocytes cultures from TB mice, a significant reduction in T-lymphocyte proliferation and both Th1 and Th2 cytokine production was observed, probably caused by a relative decrease in the number of T lymphocytes and by a lower activity. Moreover, also in ConA-stimulated whole blood, Th1 and Th2 cytokines were affected, which is probably the consequence of a general reduction in T-cell activity. These results are consistent with the immune suppression that was observed *in vivo* by the measurement of CHS. In LPS-stimulated whole blood, a significant reduction was observed on IL-1*β* and TNF-*α* production in the TB group, representing a reduced capacity of immune cells to react to an infection *ex vivo*. IL-6 showed a trend to a decrease and PGE_2_ even tended to an increase, possibly due to the high serum levels in plasma that were also present in whole blood.

After adding the complete mixture of FO, SOM and high protein/leucine to the diet of TB mice, ConA-stimulated T-cell responses were positively affected both in splenocytes and in whole blood. In addition, LPS-stimulated IL-1*β*, TNF-*α* and IL-6 production by whole blood showed a trend to an increase compared to TB mice, indicating an enhanced capacity of immune cells to mount acute infection-like responses *ex vivo.* By contrast, LPS-stimulated PGE_2_ production was decreased significantly in the TB-SNC group, which might be explained by the absence of high PGE_2_ levels in plasma or by the inhibitory effect of the complete mixture in the *ex vivo* situation.

As mentioned before, plasma levels of the pro-inflammatory cytokines IL-6 and TNF-*α* were increased significantly in cachectic TB mice, leading to a chronic inflammatory state. Together with PGE_2_, another inflammatory mediator that is enhanced significantly in TB mice, these cytokines play a central role in the induction of the immune suppression observed in these tumour-bearing mice (Young, 1994). After feeding a diet with the complete mixture of FO, SOM and high protein/leucine, IL-6 levels in plasma showed a tendency to a decrease and TNF-*α* levels were decreased significantly compared with those in the TB group. Inhibition of IL-6 production in this cachexia model is established to prevent the development of cachexia as shown after treatment with a murine antibody to IL-6 (Strassmann *et al*, 1992a), whereas TNF-*α* is thought to play an important role in wasting of fat and to induce IL-6 secretion by many cell types (Strassmann *et al*, 1992b). Nevertheless, IL-6 is not only produced by TNF-*α* signalling, it is thought that in animals carrying C26 tumours, the majority of IL-6 is produced by the tumour cells in response to macrophages residing in these tumours, leading to the severe inflammatory state inducing different catabolic processes involving cachexia (Strassmann *et al*, 1992b). Accordingly, the observed reduction in IL-6 and TNF-*α* levels in the TB-SNC group might partly account for the reduced inflammatory state and the improved functional aspects of the catabolism associated immune suppression. Also PGE_2_ might be involved in this process. PGE_2_ can be secreted by many cell types, including monocytes, macrophages and tumour cells, and suppresses multiple immune functions as T-lymphocyte proliferation and macrophage activation (Ben-Baruch, 2006). After adding the complete mixture of nutritional ingredients, PGE_2_ levels in plasma of tumour-bearing animals were reduced significantly compared with those in the TB group. This might be the result of the incorporation of the n-3 PUFA EPA (present in FO) in cell membrane phospholipids that leads, by a reduction in the percentage arachidonic acid, to a decreased production of PGE_2_ by the cyclooxygenase enzyme (Ross and Fearon, 2002; Trebble *et al*, 2003). In literature, FO has been established to possess anti-cachectic, anti-inflammatory and immune modulating properties, both *in vivo* and *ex vivo*, but dose and ratio of the n-3 PUFA EPA and DHA are described as important factors that determine these properties (Argiles, 2005; van Bokhorst-de van der Schueren, 2005; Pizato *et al*, 2006; Sijben and Calder, 2007). The FO dose of 22.1 g (providing 6.9 g EPA and 3.1 g DHA) per kg food described in the experiments with the individual nutritional ingredients (experiments A) reported nevertheless no effect on both physiological and immunological parameters, whereas the double dose tested in this model showed significant improved cachectic features and a trend to enhanced immune function measured by CHS (data not shown). In addition, no effect of adding the SOM was observed on cachexia and immune parameters (experiments A) in tumour-bearing animals, whereas a mixture of 2% (w/w) specific oligosaccharides in a 9 : 1 ratio induced Th1 immunity in an influenza vaccination model in healthy C57BL/6 mice (Vos *et al*, 2006). However, when the FO dose of 22.1 g (providing 6.9 g EPA and 3.1 g DHA) is combined with the SOM and high protein/leucine, less severe cachexia and significant improved immune responses were shown. To this end, it seems that besides inhibition of the chronic inflammatory state present in the cachectic mice, it is also required to support the nutritional state of the mice with the combination of ingredients to obtain positive effects on immune function.

In conclusion, this study showed the beneficial immune modulatory effects of the SNC, leading to a multitarget approach. No effect was observed after feeding a diet containing the individual nutritional ingredients FO, SOM or high protein/leucine on CHS in tumour-bearing animals in a pre-cachectic state. In contrast, the complete mixture of nutritional ingredients reported a significant improved Th1 immune response in tumour-bearing mice prior to weight loss. In mice already suffering from cachexia, the complete mixture of ingredients affected several physiological and immune parameters, representing a lower inflammatory state, better immune responses and less wasting of protein and lipid stores, leading to less severe cachexia. Accordingly, the combination of the nutritional ingredients FO, SOM and high protein/leucine is able to induce a synergistic effect, leading to an improved health status of the mice related to immune competence, weight gain and multiple inflammatory indices. From this, it can be concluded that the SNC has potential as immune-supporting nutritional intervention. As shown in this study, immune function in tumour-bearing mice is already affected before the onset of weight loss. Therefore, it is very important to provide nutritional support with immune-modulating properties as early as possible in order to stop or reverse the nutritional decline, slowing down the progression of cachexia and counteract dysfunction of the immune system to reduce the risk of (infectious) complications. Currently, clinical studies with cancer patients are being performed to study whether mentioned effects can be extrapolated to the human setting.

## Figures and Tables

**Table 1:** Effect of oral administration of fish oil, specific oligosaccharide mixture or high protein/leucine on physiological cachexia parameters and immune parameters

**Cachexia**	**C**	**TB**	**TB-FO**	**TB-SOM**	**TB-HPrleu**
Body weight (g)	24.4±0.3^*^	22.8±0.4	23.0±0.8	23.8±0.8	21.8±0.6
Tumour weight (g)	0.0±0.0^*^	2.2±0.1	2.1±0.1	2.2±0.1	1.8±0.1
Carcass weight (g)	24.4±0.3^*^	20.7±0.4	20.9±0.8	21.5±0.8	20.0±0.6
					
**Immune**	**C**	**TB**	**TB-FO**	**TB-SOM**	**TB-HPrleu**
Thymus weight (mg)	35.9±1.2^*^	18.7±1.0	21.1±2.1^*^	20.2±2.1	14.5±1.8
Spleen weight (mg)	98.7±2.9^*^	267.7±8.1	231.1±9.8	284.1±20.5	232.9±15.8

Data from different experiments were combined and represent means±s.e.m. of the control (C) group (*n*=40), tumour-bearing control (TB) group (*n*=40) and tumour-bearing groups after supplementation with fish oil (TB-FO, *n*=10), specific oligosaccharide mixture (TB-SOM, *n*=10) or high protein/leucine (TB-HPrleu, *n*=10).

^*^Significantly different (*P*<0.0125) from the TB group.

**Table 2:** Effects of oral administration of the complete mixture of fish oil, specific oligosaccharide mixture and high protein/leucine on physiological cachexia parameters and immune parameters

**Cachexia**	**C**	**TB**	**TB-SNC**
Body weight (g)	25.7±0.5^*^	20.1±0.4	21.9±0.5^*^
Tumour weight (g)	0.0±0.0	2.1±0.1	1.8±0.1 ^*^
Carcass weight (g)	25.7±0.5^*^	18.0±0.3	20.3±0.5^*^
Epididymus fat (mg)	230.3±17.4^*^	40.9±10.9	88.2±10.9^*^
m. Tibialis anterior (mg)	44.7±1.0^*^	33.6±0.7	38.5±0.8^*^
m. EDL (mg)	8.9±0.2^*^	6.7±0.2	7.6±0.2^*^
m. Soleus (mg)	6.4±0.2^*^	4.8±0.1	5.4±0.2^*^
m. Gastrocnemius (mg)	132.1±2.4^*^	99.5±2.2	110.7±2.9^*^
			
**Immune**	**C**	**TB**	**TB-SNC**
Thymus weight (mg)	36.8±1.8^*^	14.1±1.1	20.7±1.8^*^
Spleen weight (mg)	95.2±4.3^*^	210.5±14.3	209.8±9.3
Spleen cells (1 × 10^7^ cells/ml)	2.7±0.1^*^	5.6±0.5	5.6±0.3
Granulocytes^a^ (%)	4.6±0.6^*^	28.2±1.9	28.2±1.4
Granulocytes^a^ (cells/spleen)	50.8±7.8^*^	598.9±44.1	613.4±29.2
Monocytes^b^ (%)	2.7±0.2^*^	5.8±0.2	6.2±0.3
Monocytes^b^ (cells/spleen)	29.1±2.2^*^	126.0±8.3	139.6±11.0
Macrophages^c^ (%)	5.0±0.2	5.0±0.3	4.0±0.2^*^
Marophages^c^ (cells/spleen)	54.9±4.2^*^	110.7±10.9	87.3±5.2
CD3+CD4+ T-cells (%)	6.9±0.4^*^	3.6±0.2	4.2±0.3
CD3+CD4+ T-cells (cells/spleen)	75.7±5.8	78.7±6.3	90.8±6.2
CD3+CD8+ T-cells (%)	3.2±0.2^*^	1.7±0.1	1.7±0.1
CD3+CD8+ T-cells (cells/spleen)	35.4±3.2	34.8±2.0	36.9±2.6

Data represent means±s.e.m. of the control (C) group (*n*=10), tumour-bearing control (TB) group (*n*=19) and tumour-bearing group after oral administration of the specific nutritional combination (TB-SNC) (*n*=20).

^*^Significantly different (*P*<0.025) from the TB group.

a Defined as GR-1^high^ cells.

b Defined on the base of forward- and side-scatter profile, F4/80^dull^ and GR-1^low to dull^.

c Defined as F4/80^high^ cells.

**Table 3:** Effects of oral administration of the complete mixture of fish oil, specific oligosaccharide mixture and high protein/leucine on ConA-stimulated cytokine production in whole blood and splenocytes and on LPS-stimulated cytokine and PGE_2_ production in whole blood and splenocytes

**ConA-stimulated cytokine production**	**C**	**TB**	**TB-SNC**
*Whole blood*			
IL-2 (% *vs* C)	100.0±14.0	65.0±7.2	96.3±15.3
IL-4 (% *vs* C)	100.0±10.5^*^	11.3±3.1	69.9±23.6^*^
IL-12 (% *vs* C)	100.0±8.0	84.7±14.8	75.4±16.1
			
*Splenocytes* (*FCS*^*hi*^)			
IL-2 (% *vs* C)	100.0±11.1^*^	34.4±2.3	42.3±3.4
IL-4 (% *vs* C)	100.0±13.3^*^	45.9±4.9	70.9±11.5^*^
IFN-*γ* (% *vs* C)	100.0±10.5^*^	34.6±8.7	80.0±27.6
			
**LPS-stimulated cytokine production**	**C**	**TB**	**TB-SNC**
*Whole blood*			
IL-1*β* (% *vs* C)	100.0±8.1^*^	17.5±2.7	38.5±15.5
IL-6 (% *vs* C)	100.0±21.2	72.3±13.3	126.9±40.3
TNF-*α* (% *vs* C)	100.0±8.3^*^	18.5±2.4	38.2±15.0
PGE_2_ (% *vs* C)	100.0±8.9	137.9±20.4	43.1±3.2^*^
			
*Splenocytes* (*FCS*^*hi*^)			
IL-1*β* (% *vs* C)	100.0±4.3	107.5±11.6	97.0±13.6
IL-6 (% *vs* C)	100.0±8.6^*^	76.5±12.7	107.7±14.5
TNF-*α* (% *vs* C)	100.0±4.7	102.7±8.0	102.1±10.4
PGE_2_ (% *vs* C)	100.0±2.1^*^	178.0±12.4	196.6±11.4

ConA=concanavalin A; IL=interleukin; IFN-*γ*=Interferon-*γ*; LPS=lipopolysaccharide; PGE_2_=prostaglandin E_2_; TNF-α=tumour necrosis factor-*α*.

Data represent means±s.e.m. of control (C) group (ConA/LPS whole blood *n*=10, ConA/LPS splenocytes *n*=7), tumour-bearing control (TB) group (ConA/LPS whole blood *n*=10, ConA/LPS splenocytes *n*=13) and tumour-bearing group after oral administration of the specific nutritional combination (TB-SNC) (ConA/LPS whole blood *n*=10, ConA/LPS splenocytes *n*=12). All values were calculated as percentage of the control group, which is set at 100%. ^*^Significant differently (*P*<0.025) from tumour bearing control group (TB).
